# Ecological factors associated with dengue fever in a central highlands Province, Vietnam

**DOI:** 10.1186/1471-2334-11-172

**Published:** 2011-06-16

**Authors:** Hau V Pham, Huong TM Doan, Thao TT Phan, Nguyen N Tran Minh

**Affiliations:** 1 Institute of Hygiene and Epidemiology of Tay Nguyen, Dak Lak, Vietnam; 2 Vietnam Field Epidemiology Training Programme (FETP), Hanoi, Vietnam

## Abstract

**Background:**

Dengue is a leading cause of severe illness and hospitalization in Vietnam. This study sought to elucidate the linkage between climate factors, mosquito indices and dengue incidence.

**Methods:**

Monthly data on dengue cases and mosquito larval indices were ascertained between 2004 and 2008 in the Dak Lak province (Vietnam). Temperature, sunshine, rainfall and humidity were also recorded as monthly averages. The association between these ecological factors and dengue was assessed by the Poisson regression model with adjustment for seasonality.

**Results:**

During the study period, 3,502 cases of dengue fever were reported. Approximately 72% of cases were reported from July to October. After adjusting for seasonality, the incidence of dengue fever was significantly associated with the following factors: higher household index (risk ratio [RR]: 1.66; 95% confidence interval [CI]: 1.62-1.70 per 5% increase), higher container index (RR: 1.78; 95% CI: 1.73-1.83 per 5% increase), and higher Breteau index (RR: 1.57; 95% CI: 1.53-1.60 per 5 unit increase). The risk of dengue was also associated with elevated temperature (RR: 1.39; 95% CI: 1.25-1.55 per 2°C increase), higher humidity (RR: 1.59; 95% CI: 1.51-1.67 per 5% increase), and higher rainfall (RR: 1.13; 95% CI: 1.21-1.74 per 50 mm increase). The risk of dengue was inversely associated with duration of sunshine, the number of dengue cases being lower as the sunshine increases (RR: 0.76; 95% CI: 0.73-0.79 per 50 hours increase).

**Conclusions:**

These data suggest that indices of mosquito and climate factors are main determinants of dengue fever in Vietnam. This finding suggests that the global climate change will likely increase the burden of dengue fever infection in Vietnam, and that intensified surveillance and control of mosquito during high temperature and rainfall seasons may be an important strategy for containing the burden of dengue fever.

## Background

Dengue, including dengue fever and dengue haemorrhagic fever, is the most rapidly spreading mosquito-borne viral disease and an increasing public health problem globally [1,2]. During the past 50 years, the incidence of dengue has increased by 30 fold, parallel with the increasing geographic expansion from urban to rural areas [3,4]. According to current estimates, at least 100 countries are endemic of dengue and about 2.5 billion people are at risk in tropical and subtropical regions, with about 50 million dengue infections occurring annually [1,2]. The revised International Health Regulations 2005 included dengue as a disease that may constitute a public health emergency of international concern with implications for health security due to disruption and rapid epidemic spread beyond national borders [5].

A key strategy of the dengue control program relies on vector control to reduce viral transmission. A number of ecological, biological and social factors are involved in vector breeding and viral transmission [6]. The impact of global warming on human health, especially in vector-borne diseases, is increasingly becoming a public health concern, because the risk of dengue has been reported to be associated directly or indirectly with seasonal changes in climate [3,4,7,8], and mosquito larval indices [6,9,10].

Despite the existence of a National Dengue Control Program since 1998, dengue remains a major health problem in Vietnam. Dengue fever and dengue hemorrhagic fever are leading causes of hospitalization, accounting for 1,000,866 cases reported in Vietnam during the period 1991-2004, the highest number in the Western Pacific Region [2]. The present study was designed to describe the occurrence of dengue and its associated ecological factors in a Central Highlands province in Vietnam.

## Methods

### Study setting

**Figure 1:**
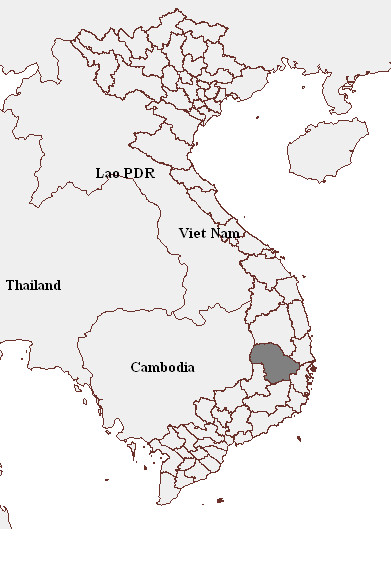
**Map of Vietnam showing Dak Lak province (in dark grey area)**.

The study was conducted in Dak Lak, a province located in the Central Highlands region of Vietnam. The region is considered one of the most disadvantaged and remote regions in the country. The province is located from 11°30'-13°25' North latitude and 107°30'-109°30' East longitudes and shares a border with Cambodia (Figure 1). Dak Lak has a relatively flat terrain with average altitude of about 500 meters above the sea level. There are two seasons: the rainy season runs from May through October with average rainfall > 100 mm, and the dry season from November through April with rainfall < 100 mm. The annual average rainfall is between 1346 and 2083 mm. Annual average temperature ranges between 22 and 26 degrees Celsius (Table 1).

**Table 1:** Climate conditions in Dak Lak province

Month	Temp. (°C)^a^	Sunshine (hours)^a^	Rainfall (mm)^a^	Relative humidity (%)^a^
Jan	21.2	241	3	79
Feb	22.6	257	1	74
Mar	24.6	270	47	73
Apr	26.0	257	78	74
May	26.0	216	194	81
Jun	25.4	202	165	84
Jul	24.2	166	237	87
Aug	24.0	145	370	88
Sep	24.0	148	434	89
Oct	23.8	171	130	86
Nov	23.0	175	74	85
Dec	21.4	169	19	83
Summary	23.9 ± 1.6 ^b^	2415 ± 109 ^c^	1751 ± 304 ^c^	81.9 ± 6.0 ^b^

**Note**:

^a ^Data are averages across 5 years (2004-2008).

^b ^Data are monthly average ± SD

^c ^Data are annual cumulative ± SD

Dak Lak has a population of 1.74 million, among whom, 30% are of ethnic minorities. It is primarily an agricultural area, with coffee plantation and production being the chief economic sector. As this is the most densely populated province in the region and it has a well established system of communicable disease surveillance, it provides an ideal setting for the investigation of dengue and its ecological correlates in the Central Highlands of Vietnam.

Dengue prevention and control activities in Dak Lak province were carried out under a multisectoral dengue action committee. Key activities included surveillance of dengue with routine weekly reports, laboratory-based sentinel surveillance, vector surveillance, and monitoring of environmental risk factors for dengue epidemics. The study was approved by Scientific Committee of Tay Nguyen University.

### Surveillance and data collection

In Dak Lak province, there is a network of 184 commune health stations located in each village. Each station is in charge of communicable disease surveillance. The data are reported first to the district's, then to the province's Center for Preventive Health.

Whenever there is a suspected case of dengue, a standard case management protocol is enacted. According to the protocol, individuals suspected to have dengue are those who meet the WHO case definition (acute febrile illness (≥38°C) of 2-7 days duration with two or more of the following non-specific manifestations of dengue fever: headache, retro-orbital pain, myalgia, arthralgia, rash, hemorrhagic manifestations, and leucopenia [1]. Once identified the patient is transferred, depending on the severity of the condition, to the nearest district hospital or provincial hospital for further diagnosis and treatment. Total numbers of dengue cases were recorded weekly during the surveillance period from 2004 through 2008.

Collection of mosquito larval indices, including the household index [HI], the container index, and the Breteau index [BI], was conducted monthly from 2004 through 2008. The collection was undertaken in accordance with guidelines recommended by the World Health Organization (WHO) [1]. During the study period, monthly surveys were carried out in 8 sites, 1 in Buon Ma Thuot City and the remaining 7 in various districts in the province. At each time, we randomly selected 8 communes from the list of communes of Buon Ma Thuot City and 7 districts. In each commune, we randomly selected a hamlet, then sampled 100 houses for data collection. The HI was the percentage of houses infested with larvae or pupae [1]. The container index was calculated as percentage of water-holding containers infested with larvae or pupae [1], and the BI was the number of positive container per 100 houses inspected [1].

Meteorological data including temperature (°C), duration of sunshine (hour), amount of rainfall (mm), and relative humidity (%) were obtained from the local meteorological offices across the province. The data represent monthly averages for each year during the study period from 2004 through 2008.

### Data analysis

The main aim of data analysis was to describe the occurrence of dengue and its association with potential ecological factors. The outcome considered in the analysis was the actual number of dengue cases which was recorded monthly during the study period for all districts of the province. Ecological factors included in the analysis were the HI, the container index, BI, temperature, duration of sunshine, amount of rainfall, and relative humidity.

As the number of reported dengue cases was small relative to the provincial population, distribution of dengue cases was assumed to follow the Poisson distribution. Accordingly, a Poisson regression model was used to model the relationships between the ecological factors and dengue cases. To control for the effect of seasonality and trend, we decomposed the incidence of dengue cases into seasonal and trend series by the classical time series method. In this method, the number of cases (Y) is decomposed into 3 components as follows: Y_t _= T_t _+ S_t _+ E_t_, where T_t _denotes the trend component, S_t _denotes the seasonal component, and E_t _is the residual component. Because the actual number of dengue cases was small relative to the population, we assumed that the incidence of dengue cases followed a Poisson distribution, with parameter λ _t_, i.e., Y_t _~ Poisson(λ _t_). The effects of ecological covariates were modeled as follows:

where *β *_t1_, *β *_t2_, *β *_t3_,...,*β *_tp _are regression coefficients associated with covariates *x*_t1_, *x*_t2_, *x*_t3_, ..., *x*_tp_, respectively. A time series Poisson regression was then fitted with weather variables, seasonal and trend components. Weather variables were included after performing seasonal difference to control for autocorrelation. First and second order autoregressive terms were considered, but only the first order regressive term was included in the final model because the second term did not reach statistical significance. All analyses were performed using the R package **gam **[11].

## Results

**Figure 2:**
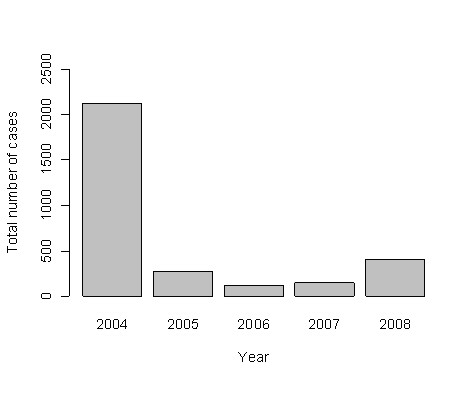
**Total number of dengue cases in Dak Lak province, 2004-2008**.

**Figure 3:**
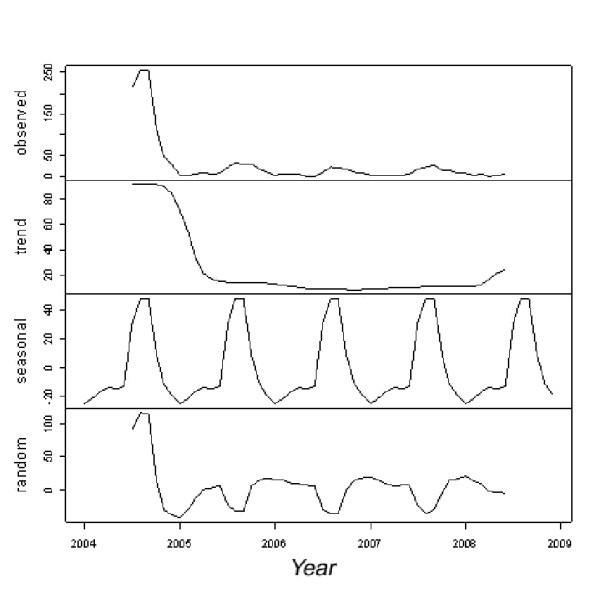
**Seasonal and trend decomposition of dengue incidence in Dak Lak province (Vietnam) between 2004 and 2008: **Top panel: monthly incidence of dengue; second panel: trend cycles of dengue incidence; third panel: seasonal cycles of dengue; and the bottom panel: random (residual) component.

During the follow-up period (2004 to 2008), 3,502 dengue cases were reported in Dak Lak province. The incidence of dengue strongly fluctuated from year to year, and between months within a year. There was an epidemic in 2004 that accounted for 71.4% of cases during the surveillance period (Figure 2). The incidence was decreased significantly after 2004. Within a year, the incidence of dengue cases peaked during the July-October period, which falls within the rainy season of May to November (Figure 3). From 2004 through 2008, the total number of dengue cases from the period of July through October accounted for 71.6% of total cases. The monthly incidence of dengue was correlated with the household index, container index, and Breteau index (Table 2). There was also a significant correlation between Breteau index, household index, and container index.

**Table 2:** Monthly mosquito indices and occurrence of dengue in Dak Lak province

Month	House Index^a^	Container Index^a^	Breteau Index^a^	No. of cases^b^
Jan	3.7	2.9	4.8	27
Feb	4.2	3.3	5.4	20
Mar	3.9	3.4	5.4	22
Apr	4.6	3.4	5.9	43
May	5.6	4.1	6.9	183
Jun	6.6	4.9	9.1	302
Jul	7.0	5.5	10.1	448
Aug	7.4	5.8	10.1	593
Sep	7.4	5.8	9.6	1007
Oct	7.1	6.2	9.7	459
Nov	5.6	3.9	6.9	281
Dec	4.7	3.2	5.7	117

**Notes**:

^a ^Data are averages across 5 years (2004-2008).

**^b ^**Data are total number of cases tallied from 2004 to 2008.

Results of univariate analysis (Table 3) showed that after adjusting for seasonality, the risk of dengue was significantly associated with increased household index, household mosquito, container index, Breteau index. Moreover, increased temperature, increased rainfall, and increased humidity were each associated with increased risk of dengue. The risk ratio ranged from 1.1 to 1.7. Because these climate and ecological variables were correlated, a multivariable Poisson regression model was fitted to the data to search for independent factors (Table 4). Results of the multivariable analysis indicated that the risk of dengue was independently associated with higher household index (RR: 1.87; 95% CI: 1.81-1.93 per 5% increase), higher mosquito index (RR: 1.08; 95% CI: 1.06-1.11 per 5% increase), increased rainfall (RR: 1.14; 95% CI: 1.12-1.15 per 50 mm), and increased temperature (RR 1.21; 95% CI: 1.11-134).

**Table 3:** Risk factors for dengue incidence in Dak Lak province: univariate analysis

Risk factor	Unit of comparison	Risk ratio (95% CI)	P-value
Household index	Per 5% increase	1.66 (1.62-1.70)	< 0.0001
Household mosquito	Per 5% increase	1.16 (1.14-1.18)	< 0.0001
Container index	Per 5% increase	1.78 (1.73-1.83)	< 0.0001
Breteau index	Per 5 unit increase	1.57 (1.53-1.60)	< 0.0001

Temperature	Per 2°C increase	1.39 (1.25-1.55)	< 0.0001
Sunshine	Per 50 hours increase	0.76 (0.73-0.79)	< 0.0001
Rainfall	Per 50 mm increase	1.13 (1.21-1.74)	< 0.0001
Humidity	Per 5% increase	1.59 (1.51-1.67)	< 0.0001

**Table 4:** Risk factors for dengue incidence in Dak Lak province: multivariate analysis

Risk factor	Unit of comparison	Risk ratio (95% CI)	P-value
Household index	Per 5% increase	1.87 (1.81-1.93)	< 0.0001
Household mosquito	Per 5% increase	1.08 (1.06-1.11)	< 0.0001

Temperature	Per 2°C increase	1.21 (1.10-1.34)	< 0.0001
Rainfall	Per 50 mm increase	1.14 (1.12-1.15)	< 0.0001

## Discussion

Previous studies have shown that the dynamics of dengue is affected by many factors, including environmental and climate factors, host-vector interactions and the herd immunity [12-15]. Climate factors may directly or indirectly affect vector survival, lifespan, development and reproductive rates that could influence dengue spatio-temporal distributions [7,16-19]. The present study demonstrated a clear seasonal pattern of dengue occurrence in a Central Highlands province of Vietnam, with the highest number of cases occurring in the rainy season. Elevated temperature, relative humidity and duration of sunshine were also associated with an increased occurrence of dengue.

Our result is consistent with previous studies which found that the majority of dengue cases occurred in months with higher rainfall that were clearly defined "dengue season" [8,20-22]. In Trinidad, "dengue season" was between June and November [23], and evidence showed that the dengue incidence in Metro Manila varies with changing rainfall patterns [24]. It has been hypothesized that rainfall affects adult female mosquito density. An increase in amount of rainfall leads to more breeding sites which, in turn, lead to an increase in the number of mosquitoes. An increase in the number of adult female mosquitoes increases the probability of viral transmission [25]. Elevated temperature is associated with an increased incidence of dengue due to accelerated development rate of the virus and increased mosquito biting rate [26], as well as increased development rate of different mosquito life stages and dengue virus replication. Higher temperature enhances virus replication and shorten the extrinsic incubation period in the vector [27] whereas higher relative humidity decreases adult mosquito mortality [21].

In Dak Lak province, we found that the rainy season is characterized by a higher BI, container index, and HI, which is also consistent with a previous observation that a positive associations between the incidence of dengue and the *Aedes *HI and the BI [21,28]. This relationship suggests that the latter might have exerted its effect on dengue infection partly through the creation of more breeding sites for *A. aegypti*. Surveillance by ovitrap (a device consisting of a black painted milk can, filled with 250 ml tap water and a hole each side of tin) in a selected urban area and suburban area in Malaysia for 14 months found a strong correlation between rainfall and egg population [29]. A retrospective ecological study on impact of weather variables and climatic indicators associated with the incidence of dengue conducted in Mexico from 1995 to 2003 showed that increases in weekly minimum temperature and rainfall were also significant factors in the increase in the reported cases of dengue [30]. A study in Barbados has documented a correlation between the incidence of dengue in a specific parish from 1995-2000, and a range of climate variables, with lags from 7-16 weeks [31]. A study in 14 provinces of southern Thailand indicated that the temperature, rainfall and humidity were associated with dengue hemorrhagic fever incidence [32].

The present study should be interpreted within the context of strengths and limitations. In the province, health workers at all levels are very familiar with dengue fever and dengue hemorrhagic fever, as proven by the low case-fatality rate [2] and the standard clinical case definition for dengue has been used for more than 10 years without substantial change [1]. Dengue diagnosis is based on standard clinical case definition of WHO for identifying outbreak and initiating an early response as well as reducing dengue mortality. However, a recent study on dengue diagnosis using WHO's standard clinical case definition for dengue showed high sensitivity but poor specificity [33]. Therefore, there existed a possibility of overestimation of the number of dengue cases in the study. However, to our knowledge, there were a number of dengue patients treated in private health services that could not recorded. Indeed. a study of diagnosis of acute undifferentiated fever in Vietnam showed that acute dengue was found in ~34% cases, which suggests that the possibility of underreporting of dengue in commune health stations [34]. The present study was an ecological investigation; therefore, it is not possible to make inference concerning the causative relationship between the mosquito larval indices and dengue infection at the individual patient level. The number of deaths was small and therefore not suitable for a thorough analysis of risk factors for dengue mortality.

## Conclusions

In summary, our study in the Central Highlands of Vietnam showed that the risk of dengue increased during rainy months when monthly rainfall increased together with the increased vector population as indicated by the monthly HI, container index and BI. The global climate change will likely increase the burden of dengue fever infection in Vietnam. Intensified surveillance and control of mosquito during high temperature and rainfall seasons may be an effective strategy for containing the burden of dengue fever.

## Competing interests

The authors declare that they have no competing interests.

## Authors' contributions

HVP and HTMD designed the study, performed data analysis, interpretation and drafted the manuscript. HVP, HTMD and TTTP contributed to the collection, entry, analysis and interpretation of data. HVP and NNTM performed data analysis, interpretation and wrote the manuscript. All authors read and approved the final version of the manuscript.

## Pre-publication history

The pre-publication history for this paper can be accessed here:

http://www.biomedcentral.com/1471-2334/11/172/prepub
